# Machine Learning-Based Gully Erosion Susceptibility Mapping: A Case Study of Eastern India

**DOI:** 10.3390/s20051313

**Published:** 2020-02-28

**Authors:** Sunil Saha, Jagabandhu Roy, Alireza Arabameri, Thomas Blaschke, Dieu Tien Bui

**Affiliations:** 1 Department of Geography, University of Gour Banga, Malda, West Bengal 732103, India; sunilgeog@ugb.ac.in; 2 Research Scholar, Dept. of Geography, University of Gour Banga, Malda, West Bengal 732103, India; jagabandhuroy1991@gmail.com; 3 Department of Geomorphology, Tarbiat Modares University, Tehran 14117-13116, Iran; 4 Department of Geoinformatics—Z_GIS, University of Salzburg, 5020 Salzburg, Austria; thomas.blaschke@sbg.ac.at; 5 Institute of Research and Development, Duy Tan University, Da Nang 550000, Vietnam

**Keywords:** random forest (RF), gradient boosted regression tree (GBRT), tree ensemble (TE), Naïve Bayes tree (NBT), R programming language, geographical information system (GIS)

## Abstract

Gully erosion is a form of natural disaster and one of the land loss mechanisms causing severe problems worldwide. This study aims to delineate the areas with the most severe gully erosion susceptibility (GES) using the machine learning techniques Random Forest (RF), Gradient Boosted Regression Tree (GBRT), Naïve Bayes Tree (NBT), and Tree Ensemble (TE). The gully inventory map (GIM) consists of 120 gullies. Of the 120 gullies, 84 gullies (70%) were used for training and 36 gullies (30%) were used to validate the models. Fourteen gully conditioning factors (GCFs) were used for GES modeling and the relationships between the GCFs and gully erosion was assessed using the weight-of-evidence (WofE) model. The GES maps were prepared using RF, GBRT, NBT, and TE and were validated using area under the receiver operating characteristic (AUROC) curve, the seed cell area index (SCAI) and five statistical measures including precision (PPV), false discovery rate (FDR), accuracy, mean absolute error (MAE), and root mean squared error (RMSE). Nearly 7% of the basin has high to very high susceptibility for gully erosion. Validation results proved the excellent ability of these models to predict the GES. Of the analyzed models, the RF (AUROC = 0.96, PPV = 1.00, FDR = 0.00, accuracy = 0.87, MAE = 0.11, RMSE = 0.19 for validation dataset) is accurate enough for modeling and better suited for GES modeling than the other models. Therefore, the RF model can be used to model the GES areas not only in this river basin but also in other areas with the same geo-environmental conditions.

## 1. Introduction

One of the major problems in modern societies in the last decade is the degradation of natural resources, especially soil and water [1]. The rapid population growth and careless use of natural resources lead to soil and water degradation, which in turn threatens human lives and property [2,3].

Soil erosion by water, such as in the form of gully erosion, is one of the most common soil degradation processes worldwide [4,5]. Gully erosion typically features a deep channel eroded by running surface water that removes and transports the eroded surface soil particles and other materials [6]. Gully erosion causes various environmental problems like desertification, inundation, and sedimentation in lakes [6,7], as well as reducing the soil fertility and agricultural productivity, which negatively affects the economy [8,9]. When water erosion or sediment formation exceeds the geomorphological threshold value of an area, then the process of gully erosion occurs [10]. Gully erosion mapping is essential for implementing soil conservation initiatives [6]. Geo-environmental factors such as precipitation, altitude, slope, aspect, curvature of the plane, lithology [11], soil physio-chemical properties [12], and land use/land cover (LULC) [13] have a strong influence on gully erosion. 

A gully erosion susceptibility map (GESM) is obtained using the relationship between gully occurrence and geo-environmental gully conditioning factors [11]. To calculate the rate of soil erosion, various numerical and conventional methods were applied, such as the universal soil loss equation (USLE) [1,14], the potential erosion process, the Modified Southwest Interagency Committee Model (MPSIAC), the water erosion project (WEEP) [15], the European Soil Erosion Model (EUROSEM) [16] etc. In the last two decades, the use of remote sensing data to predict gully susceptibility has increased enormously [17]. Presently, combined with remote sensing and GIS, different probabilistic, knowledge-driven and machine learning methods are being used to generate GESM, such as bivariate statistics (BS) [1], weights-of-evidence (WoE) [18,19,20], logistic regression (LR) [21,22,23,24], information value (IV) [22], random forest (RF) [25], bivariate statistical models [26], maximum entropy (ME) [27], frequency ratio (FR) [26,28], analytical hierarchy processes (AHP) [29], artificial neural network (ANN) [11,27], Functional tree (FT), Naïve Bayes tree (NBTree) [12], support vector machine (SVM) [27] and boosted regression trees (BRT) [11]. In the present research, tree-based machine learning algorithms, namely the Random Forest (RF), Gradient Boosted Regression Tree (GBRT), Naïve Bayes Tree (NBTree), and Tree Ensemble (TE) models were used to model the gully erosion susceptibility. The RF model is controlled by machine learning algorithms that use multiple trees in the classification [17]. The RF method uses large numbers of decision trees to consider the factors or variables affecting the target variable. The RF algorithm then combines all the trees to make decisions [17]. Tree-based machine learning methods have been used for gully erosion modeling by various researchers who have stated that the RF, BRT, naïve Bayes tree (NBTree), and Functional trees (FT) have shown better performance and precision for gully erosion susceptibility GES modeling than conventional methods [12,30]. The overfitting problem in such tree-based machine learning methods is very low compared with the numerical models [31,32].

The integration of GIS and the R programming language has provided the best platform for preparing more accurate susceptibility models. Arabameri et al. [30], Hosseinalizadeh et al. [12] used GIS and the R programming language in spatial gully erosion modeling, and they found the combined method to be more accurate than conventional methods. R programming language-based machine learning methods are more reliable and accurate [12,13,27,30]. Viewing the better accuracy of the machine learning models used in other fields than the conventional, knowledge-driven, probabilistic models as per the previous literature, we selected the four trees-based machine learning techniques for predicting the spatial susceptibility of gully erosion in the Hinglo River basin in eastern India. The Tree Ensemble (TE) method, which we have selected for gully erosion prediction, is a new method and has previously not been used for hazard mapping. The selected machine learning methods were used to prepare gully erosion susceptibility maps of the study area and the results were compared.

In the upper and upper-middle catchment areas, the Hinglo River basin is currently subject to gully erosion. For this reason, it is necessary to address the problem of soil erosion in the study area. Therefore, the main objective of this study is to ascertain which areas are susceptible to gully erosion using machine learning ensemble techniques, namely RF, GBRT, Tree Ensemble (TE), and Naïve Bayes Tree (NBT), and compare the results. These gully erosion susceptibility models will help the agricultural planners to predict the probability of soil erosion for better land management.

## 2. Materials and Methods

### 2.1. Description of The Study Area

**Figure 1:**
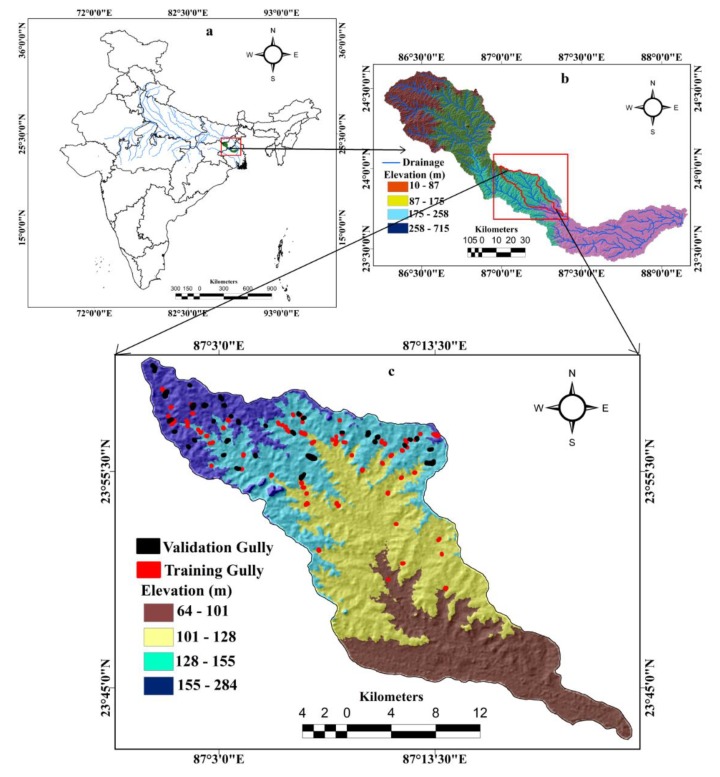
Study area showing (**a**) India, (**b**) Ajay River Basin, (**c**) Hinglo River Basin.

The Hinglo River basin geographically extends from 23°42′7.09″ N to 24°0′56.78″ N latitude and 86°59′32.68″ E to 87°23′31.91″ E longitude (Figure 1). The total catchment area is 442.95 km^2^. The Hinglo River basin, which is a major tributary of the Ajay river basin, encompasses part of the Jamtra district and the Birbhum district in India. The length of the primary river is 66 km. Physiographically, most of this basin is part of the Chota Nagpur plateau fringe region. The study area is subjected to the Indian monsoon climate, with an average annual rainfall of 1316–1361 mm. This basin encompasses five geological formations, namely granite-gneiss, barker, ironstone shale, newer alluvium, and quartzite [33]. The alluvium thickness of the eastern part of the basin varies between 12 and 20 m [34]. The depth of the groundwater table of this region varies between 5 and 10 m b.g.l [35]. The area has seven soil texture classes, namely sand, clay, clay loam, haplustepts, sandy loam, loam, and fine loamy [36], whereby haplustepts covers most of the basin. The maximum elevation of the study is 284 m a.s.l. The north and middle-western parts of this study area are subjected to gully erosion [37]. A precise gully erosion susceptibility map is essential for this region to manage the erosion-prone areas.

### 2.2. Methodology

Different types of data were collected from various sources to fulfill the intent of our research (Table 1). The gully locations were identified from field investigation using a handheld global positioning system (GPS) and Google Earth image. Table 1 details the data used in this study.

**Figure 2:**
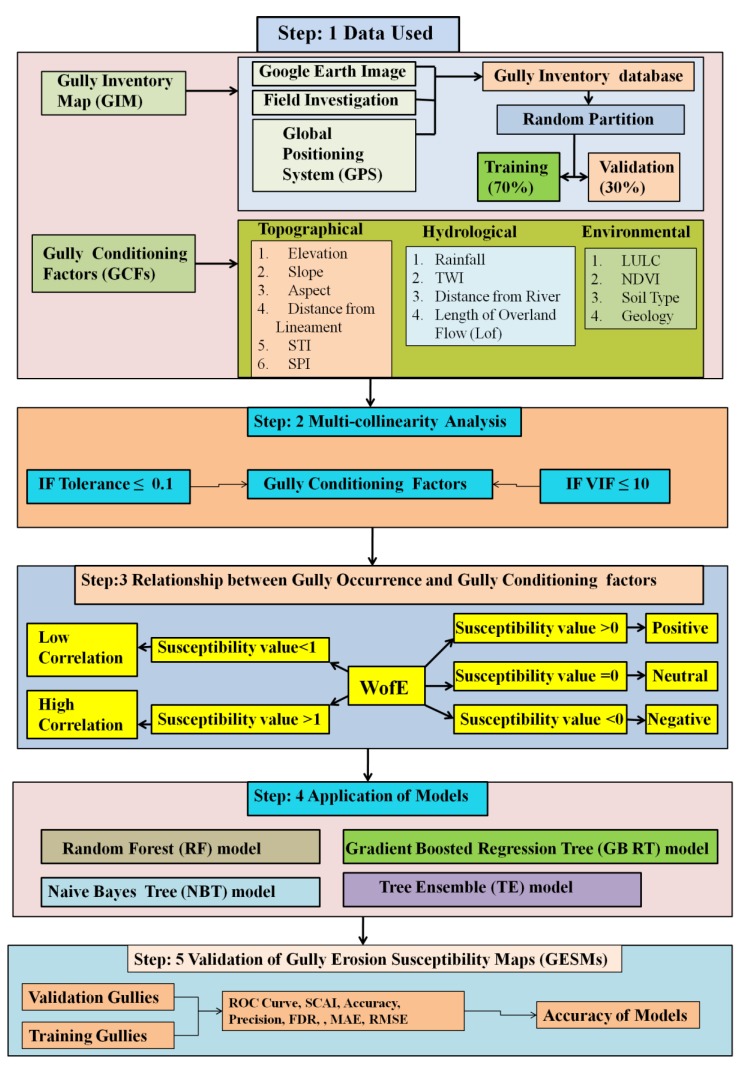
Flowchart showing the methodology of the present work.

The present study was carried out in the following five main steps (Figure 2): (1) Gully inventory map (GIM) and GCFs data layers were prepared; (2) a multi-collinearity analysis was carried out to select the gully erosion conditioning factors (GCFs); (3) the weight-of-evidence (WofE) method was used to examine the relationship between gully erosion and GCFs; (4) the machine learning models RF, NBT, GBRT, and TE models were applied to prepare the GESMs, and (5) the performance of each of the ensemble models was evaluated using the area under the receiver operating characteristic (AUROC) and SCAI methods and a few statistical measures. 

### 2.3. Database

#### 2.3.1. Preparing the Gully Inventory Map (GIM)

**Figure 3:**
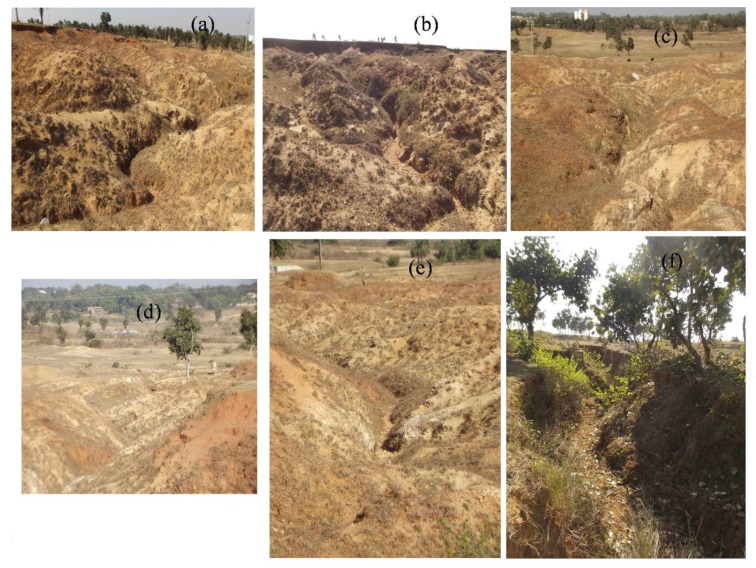
Field photographs of gullies of fallow land and vegetation-covered areas (**a**) Charakmara (24°00′36″ N, 86°54′48″ E), (**b**) Dhainghati (23°57′56″ N, 87°00′34″ E), (**c**) Agaia (23°57′15″ N, 87°10′14″ E), (**d**) Bamombhuin (23°56′41″ N, 87°12′07″ E), (**e**) Hesaltanr (23°56′13″ N, 87°07′31″ E), (**f**) Prasadpur (23°57′03″ N, 87°12′36″ E)

The GIM is essential for preparing the GESMs by various predictive models [27] and was considered as the dependent variable in this study area. To prepare the GIM, first, gully locations and dimensions were measured using the remotely sensed data through Google Earth. Then, a field investigation was conducted in the study area to update and ground truth check the data. Gully locations were geolocated with handheld GPS. A total of 120 gullies were identified in the study area. Of the 120 gullies, 84 (70%) gullies were randomly selected for model preparation, and the remaining 36 (30%) gullies were used for model validation (Figure 3) based on previous literature [20,23,24]. Representative gully images are shown in Figure 1.

#### 2.3.2. Preparing the Gully Conditioning Factors (GCFs)

Selecting geo-environmental factors is an important step in preparing the GESMs using various methods [11]. In this study, 14 GCFs, namely elevation, slope, aspect, monsoonal rainfall, soil type, geology, LULC, NDVI, distance to river, distance to lineament, Lof, TWI, STI, STI were used for spatial gully erosion modeling while considering the previous literature and multi-collinearity analysis. 

**Figure 4:**
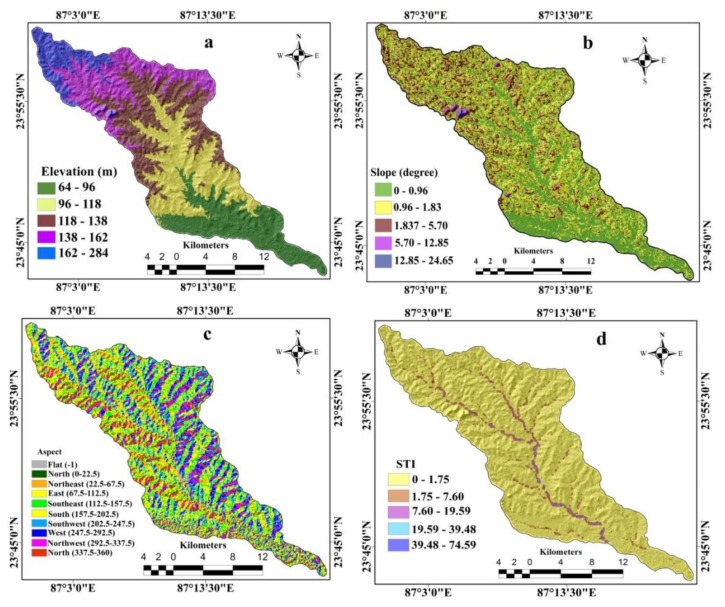

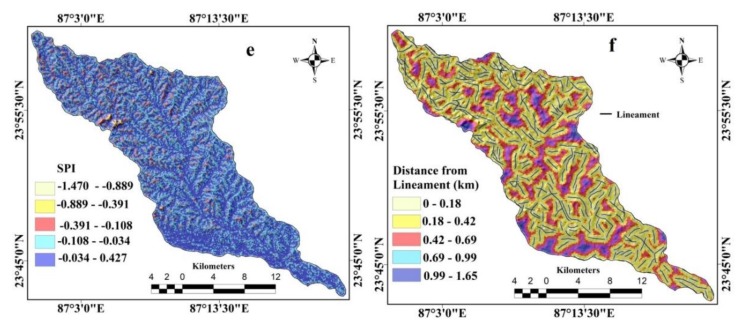
The topographical factors: (**a**) elevation, (**b**) slope, (**c**) aspect, (**d**) sediment transportation index (STI), (**e**) stream power index (SPI), (**f**) distance from lineament.

The digital elevation model (DEM), collected from USGS, was used as the elevation data layer (Figure 4a). The altitude of the study area was categorized into five classes, namely 64 m–96 m, 96 m–118 m, 118 m–138 m, 138 m–162 m, 162 m–284 m (Figure 4a). The slope affects gully erosion significantly [26]. The slope map was prepared in GIS from a recorded DEM (Figure 4b) and was classified into the five classes of 0–0.96, 0.96–1.83, 1.83–5.70, 5.70–12.85, 12.85–24.65 (Figure 4b). Like the slope map, the aspect map was derived from the DEM (Figure 4c) and divided into nine subgroups: flat (−1), north (0–22.5, 337.5–360), north-east (22.5–67.5), east (67.5–112.5), south-east (112.5–157.5), south (157.5–202.5), south-west (202.5–247.5), west (247.5–292.5), north-west (292.5–337.5) (Figure 4c). The sediment transportation index (STI) was calculated using Equation (Equation (1)) suggested by Moore and Burch [38], and it was also derived from the DEM.

(1) $$\mathrm{STI}=(M+1)\times (\mathrm{As}/22.13{)}^{m}\times \sin(B/0.0986{)}^{n}$$

where “As” is the area of a specific catchment; “B” is the slope gradient in degrees; m is constant, i.e., 0.4, “n” is constant, i.e., 0.0896. The STI was classified into the five classes of 0–1.75, 1.75–7.60, 7.60–19.59, 19.59–39.48, 39.48–74.59 (Figure 4d). The SPI reflects the discharge, carrying capacity, and runoff erosion power, which determines the gully erosion susceptibility [22,39,40]. The SPI was derived from DEM using the following Equation (2).

(2) $${\mathrm{SPI}=A}_{S}\times \tan\beta$$

where A_S_ is the upstream contributing area and β is slope gradient (in degrees). The SPI was categorized into the five classes of −1.470 to −0.889, −0.889 to −0.391, −0.391 to −0.108, −0.108 to −0.034 and −0.034 to 0.427 (Figure 4e). Using GEOMATICA and ENVI 4.7 software, the lineament of the study area was derived from the Landsat 8 OLI / TIRS panchromatic band. The distance to lineament map was built using the (Figure 4f) EDB tool in GIS. The lineament buffer was classified into the classes of 0–0.18 km, 0.18–0.42 km, 0.42–0.69 km, 0.69–0.99 km, and 0.99–1.65 km distance (Figure 4f).

**Figure 5:**
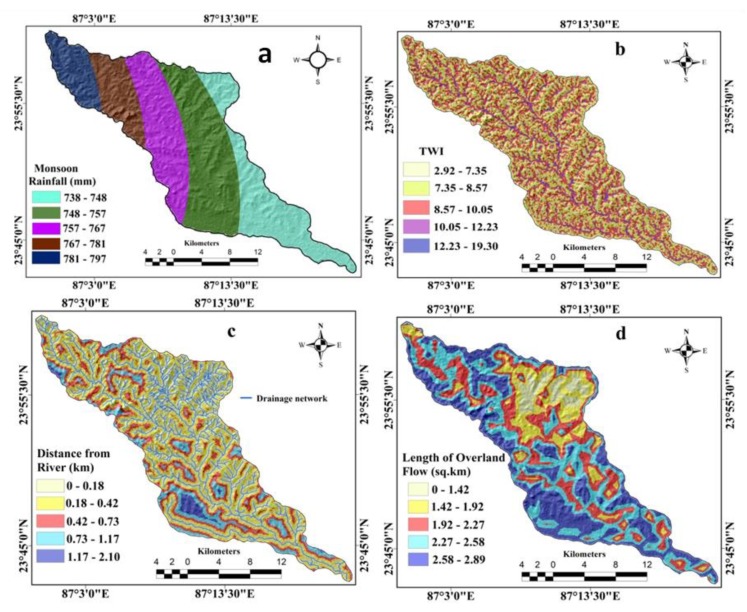
Hydrological factors: (**a**) monsoonal rainfall, (**b**) topographical wetness index (TWI), (**c**) distance to river, (**d**) length of overland flow (Lof).

The study area’s average monsoonal rainfall map was prepared using the kriging method based on the rainfall data of the last three years measured at different stations. The monsoonal rainfall was categorized into the five sub-classes of 738–748, 748–757, 757–767, 767–781, 781–797 (Figure 5a). The TWI was defined by Beven and Kirkby [41]. It is commonly used to evaluate a region’s hydrological features [42]. The TWI is considered to be an important gully erosion determining factor [39]. The TWI was derived from DEM imagery using the following Equation (3).

(3) $${\mathrm{TWI}=\mathrm{In}\;(A}_{S}/\tan\beta )$$

where A_S_ is the upstream contributing area and β is the slope gradient (in degrees). The TWI was classified into five sub-categories, namely 2.92–7.35, 7.35–8.57, 8.57–10.05, 10.05–12.23, and 12.23–19.30 (Figure 5b). The distance from the river map was prepared by applying the Euclidian distance buffer (EDB) tool in GIS (Figure 5c). It was categorized into five sub-classes, namely 0–0.18 km, 0.18–0.42 km, 0.42–0.73 km, 0.73–1.17 km, and 1.17–2.10 km distance (Figure 5c). 

The measured length of the overland flow (Lof) was introduced by Horton [43] and is calculated using Equation (4).

(4) $$\mathrm{Lof}=\frac{1}{2\mathrm{Dd}}$$

where Dd is the drainage density. Drainage density is the total length of stream per unit area. The Lof was categorized into five sub-classes, namely 0–1.42 km^2^, 1.42–1.92 km^2^, 1.92–2.27 km^2^, 2.27–2.58 km^2^, and 2.58–2.89 km^2^ (Figure 5d).

**Figure 6:**
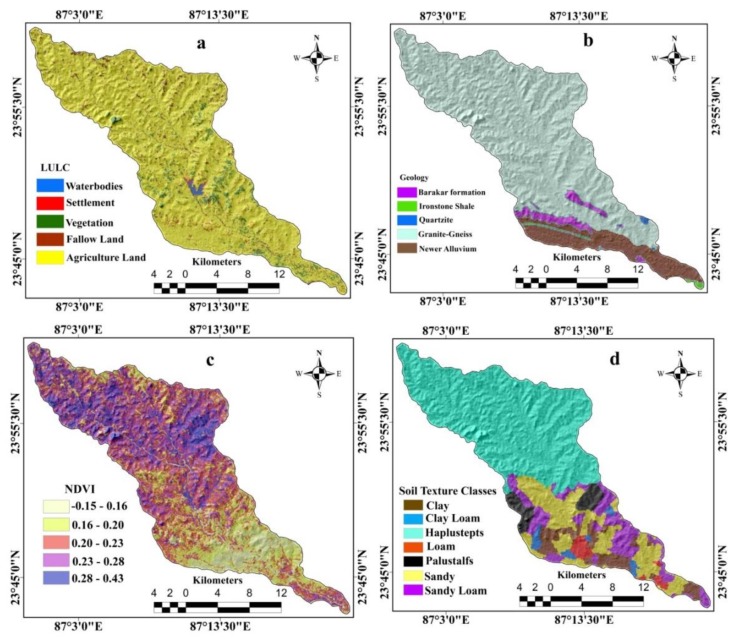
The environmental factors: (**a**) land use/land cover (LULC), (**b**) geology, (**c**) normalized difference vegetation index (NDVI), (**d**) soil type.

The LULC map was extracted from Landsat 8OLI/TIRS imagery based on the maximum likelihood classification method in GIS (Figure 6a). Water bodies, fallow land, agricultural land, settlement, and natural vegetation are the land use types found in the basin (Figure 6a). Using the digitization process in GIS environment, the geological map was generated for the study area (Figure 6b). Geologically, the study area consists of five geological formations, i.e., iron shale, barakar formation (comprises several meters of thick pebbly or conglomeratic succeeded by heterolithic cross-stratified sandstone–mudstone–carbonaceous shale–coal beds), quartzite, granite-gneiss, and newer alluvium (Figure 6b). The NDVI map was prepared using the Landsat 8OLI/TIRS imagery in a GIS environment (Figure 6c) with the help of Equation (5).

(5) $$NDVI=\frac{IR-R}{IR+R}$$

where *IR* is the electromagnetic spectrum’s infrared portion, and *R* is the electromagnetic spectrum’s red portion. The NDVI was classified into five classes, namely −0.15 to 0.16, 0.16 to 0.20, 0.20 to 0.23, 0.23 to 0.28, and 0.28 to 0.43 (Figure 6c). The soil type map was prepared using the district’s registered soil type map in GIS (Figure 6d). Pedologically, the study area is composed of seven soil texture classes namely clay, fine loamy mixed (Haplustepts), clay loam, loam, fine loamy mixed type palustepts, sand and sandy loam (Figure 6d).

In this study, the elevation, slope, rainfall, distance from river, distance from lineament, NDVI, TWI, SPI, and STI were used as numerical variables and reclassified into five sub-categories using the NBM in GIS. The aspect, geology, soil type, land use/land cover were used as categorical variables. The presence and absence of gullies were used as target variables.

### 2.4. Multi-Collinearity Analysis of Effective Factors

The multi-collinearity test is an important way to judge the linear dependency among the selected independent factors in the statistical modeling [44]. In the case of the machine learning models, this technique needs to be used for better results [45,46,47,48,49,50,51,52]. Researchers have applied multi-collinearity analysis for gully erosion susceptibility mapping [53], groundwater potentiality mapping [54], landslide susceptibility mapping [48] etc. The multi-collinearity was tested using the tolerance (TOL) and variance inflation factor (VIF). The TOL was calculated using Equation (6), where R2 is obtained by the regression of each variable for the remaining variables in the multivariate regression [55].

(6) $$TOL=1-R_{j}^{2}$$

(7) $$VIF=\frac{1}{TOL}$$

where *R*^2^*j* is the regression value of explanatory *j* on all other independent variables. A tolerance of less than 0.10 and a VIF value of 10 and above indicate a multi-collinearity problem [56].

### 2.5. Assessment of The Relationship between Gully Erosion and Effective Factors using Weight-of-Evidence (WofE) Model

The WofE is an important bivariate statistical method, which calculates the relative importance of effective factors by statistical means using the log-linear form of the Bayesian probability model [57]. In this analysis, the WofE model was used to demonstrate the relationship between gully occurrence and gully conditioning factors [51] obtained using the regression of each variable [55].

(8) $$X_{i}^{+}=\log_{e}\left[\left(B_{pix1}/(B_{pix1}+B_{pix2}))/(B_{pix3}/(B_{pix3}+B_{pix4})\right)\right]$$

(9) $$Y_{i}^{-}=\log_{e}\left[\left(B_{pix2}/(B_{pix1}+B_{pix2}))/(B_{pix4}/(B_{pix3}+B_{pix4})\right)\right]$$

where *B*_*pix*1_ is the number of pixels of gully erosion in a particular class, *B*_*pix*2_ is the total number of pixels of gully erosion on a map, *B*_*pix*3_ is the number of pixels in a specific class of GCF, and *B*_*pix*4_ is the total number of pixels in a map. A is positive weight $X_{i}^{+}$ indicates the existence of a gully pixel and a positive relationship between the presence of the gully pixel and GCF and vice versa. Finally, the weight was calculated using Equation (10) [26,58].

(10) $$F=\left(\frac{P}{Q(P)}\right)$$

where, *F* is the weight, and *P* is the differential weight between positive and negative. *P* is negative for a negative correlation and positive for a positive correlation between GCFs and gully erosion [59]. *Q* (*P*) is the standard deviation (SD) of the weight contrast.

### 2.6. Models for Spatial Gully Erosion Mapping

#### 2.6.1. Random Forest (RF) Model

Decision trees were used to generate subset training datasets for the preparation of the final model based on the random sampling method [60]. The T (number of trees) and m (number of variables) are the important features of the RF model and are defined by the user. Micheletti et al. [60] concluded that a calibration set is not essential for defining the parameters. Calle and Urrea [61] noted that the RF model could be used for analyzing the importance of the factors. In this analysis, the RF model consists of the two trees (presence and absence of gullies) that are evaluated by the 14 random independent variables. For the RF algorithm, the generalization error is measured as follows [62].

(11) $$GE=P_{x,y}(mg(x,y)<0)$$

(12) $$mg(x,y)=av_{k}I(h_{k}(x)=y)-\max_{j\neq k}av_{k}I(h_{k}(x)=j))$$

where *x* and *y* represent the contributing factors to gully erosion displaying probabilities over *x* and the margin function, and the indicator function are represented by *y* space, *mg* and I (*) [63].

#### 2.6.2. Naïve Bayes Tree (NBT) Model

Kohavi [64] suggested the use of the Naïve Bayes tree (NBT) method, a hybrid algorithm of decision tree and Naïve Bayes. The NBT model uses very little training data to evaluate the most important modeling and classification parameters [65]. The NBT was used as the reference classifier to evaluate the vulnerability to gully erosion in an ensemble framework [66]. The NBT operates as follows [67].

(13) $$t_{NB}=\arg\max_{Zi}PP(t_{i})\prod_{i=1}^{m}\frac{1}{\sqrt{2\pi \varepsilon }}e^{\frac{-{\left(r_{i}-\sigma \right)}^{2}}{2\varepsilon^{2}}}$$

where *pp(t_i_)* is the earlier variables output probability *t_i_* = (1,0), σ and ε indicate the average and SD of *r_i_* respectively

#### 2.6.3. Gradient Boosting Regression Tree (GBRT)

The GBRT was introduced by Friedman [32] and is an important machine learning technique. Boosting is a popular learning approach that was specifically designed to overcome categorization issues but has also been effectively extended to regression. The impetus for boosting is to unite a powerful committee with the output of many weekly learners [68]. The works of Hastie et al. [68], Ridgeway [69] and Scikit-learn [70] provide an in-depth description of gradient boosting and gradient boosted regression trees (Algorithm 1).
**Algorithm 1.** Gradient Boosting Regression Tree (GBRT).$\begin{array}{l} 1.\;F_{0}\left(x\right)=\arg\min_{p}\sum_{i=1}^{N}L\left(y_{i,}\rho \right)\\ 2.\;\mathrm{For}\;m=1\;\mathrm{to}\;M\;\mathrm{do};\\ 3.{\bar{\;y\;}}_{i}=-{\left[\frac{\partial L(y_{i,}F(x_{i}))}{\partial F(x_{i})}\right]}_{F(x)=F_{m-1}(x)},i=1,N\\ 4{.\;a}_{m}=\arg\min_{a}\beta {\sum_{i}^{N}\left[{\bar{y}}_{i}-\beta h(x_{i};a)\right]}^{2}\\ 5.\;\rho_{m}=\arg\min_{\rho }\sum_{i=1}^{N}L(y_{i,}F_{m-1}\left(x_{i}\right)+\rho h(x_{i};a_{m}))\\ 6.\;F_{m}(x)=F_{m-1}(x)+\rho_{m}h(x;a_{m})\\ 7.\;\mathrm{end}\;\mathrm{Forr}\\ 8.\mathrm{end}\;\mathrm{Algorithm} \end{array}$

These algorithms have been considered for different prediction purposes as found in the literature of Persson et al. [31], Friedman [32], Hastie et al. [61], Ridgeway [69], and Scikit-learn [70].

#### 2.6.4. Tree Ensemble (TE) Model

The Tree Ensemble method combines various decision tree models to produce a more suitable and accurate predictive model than using a single tree model. The TE method consists of two decision tree methods, such as bagging and boosting. The random forest model uses the bagging and the gradient boosted regression tree model uses the boosting method. Therefore, the TE method is the sum of the ensemble of all tree models [71]. A sum-ensemble of trees model $f:{\mathbb{R}}^{2}\to \mathbb{R}$ consists of a Ʈ of regression trees. Keeping the generality unchanged, a regression tree *T* ∈ Ʈ is a binary tree where each internal node n∈t.nodes bears a rational predicate over the feature variables. The prediction of tree *T* is the leaf value of the prediction path. Finally, the signed margin prediction *f(x)* of the ensemble model is the sum of predictions of all individual trees, and the predicted label is acquired by the threshold value generally set at zero:c(x)=1⇔f(x)>0.

In this study, we consider the case of single-feature threshold predicates of the form $x_{i}<T$ or equivalently $x_{i}>T$ where 0≤i<n and T∈ℝ fixed model parameters. This restriction keeps out oblique decision trees where predicates concurrently engage numerous feature variables. However, we note that oblique trees are seldom used in ensemble classifiers, partially because of their relatively high construction cost and complexity [72]. Kantchelian et al. [71] have used the equation and technique of the TE model for evasion and hardening of tree ensemble classifiers. Xiao et al. [73] have also used the tree ensemble classifier technique for identifying the different transportation nodes.

### 2.7. Validation Methods

In the present study, we used five statistical measures, namely precision (PPV), false discovery rate (FDR), accuracy, mean absolute error (MAE), and root mean squared error (RMSE) to evaluate the robustness of the used machine learning ensemble models. PPV is the proportion of units with an expected positive outcome that is positive for the true condition (Equation (14)). FDR is the proportion of the units with a predicted positive condition for which the true condition is negative (Equation (15)). The accuracy represents the maximum proportion of accurately estimated or defined units (Equation (16)). The MAE (Equation (17)) and RMSE (Equation (18)) were used to measure the variation between observed and predicted data. The robustness of models is good when PPV and accuracy are high, and FDR, MAE, and MRSE are low [74,75,76].

(14) $$PPV=\frac{A}{\left(A+B\right)}$$

(15) $$FDR=\frac{B}{\left(A+B\right)}$$

(16) $$Accuracy=\frac{A+D}{\left(A+B+C+D\right)}$$

(17) $$MAE=\frac{1}{n}\sum_{i=1}^{n}\left|X_{predicted}-X_{actual}\right|$$

(18) $$RMSE=\sqrt{\frac{1}{n}\sum_{i=1}^{n}{\left(X_{predicted}-X_{actual}\right)}^{2}}$$

A standard tool for evaluating the model performance is area under the receiver operating characteristic (AUROC) curve [77,78]. ROC is plotted on the x- and y-axis based on the sensitivity and 10-specificity. The model output was assessed using the AUC (area under the curve) of ROC (Equation (19)) [77,78]. In the previous studies [79], the mathematical theory and equation of this approach are fully described. The sensitivity (i.e., probability detection) addresses the question of which part of the detected gullies is labeled accurately and its optimal value is 1 [80]. The specificity (i.e., negative predictive value) addresses the question of which part of the non-gullies is categorized correctly, and its optimal value is 1. The AUC values below 0.6, 0.6–0.7, 07–0.8, 0.8–0.9, and above 0.9 indicate a bad, medium, decent, very good, and excellent quality of the model. The training data set’s ROC indicates the model’s success rate and tests the model’s suitability [81]. The test dataset’s ROC reveals the model’s predictive value and shows how good or bad the predictive model [79] is. The seed cell area index (SCAI) is an important method for judging the robustness of the models [24,82] and was used in this study.

(19) $$\mathrm{AUROC}=\frac{\sum A+\sum D}{A+D+B+C}$$

where *A* is the true positive rate, *B* is the false positive, *C* is the false negative, *D* is the true negative.

## 3. Results 

### 3.1. Analysis of Muti-Collinearity of GCFs

The multi-collinearity indicates the intra-correlation among the gully conditioning factors. The multi-collinearity test was carried out by the SPSS software. The outcomes of the multi-collinearity between the 14 GCFs are presented in Table 2. The results of the multi-collinearity show that the tolerance and VIF values of gully conditioning factors are less than 0.1 and 4.5, indicating no multi-collinearity problems among the gully conditioning factors, which means that they can be used for predicting the gully erosion.

### 3.2. Analysis of Factor Importance using the Weight-of-Evidence (WofE) Model

The statistical calculation of the WofE model is shown in Table 3. The WofE values of independent factors represent their effect on gully development. The topographic factors like elevation, slope, and aspect are strongly related to gully erosion. When the WofE value is >1 the control of the effective factor on gully occurrence is high and vice versa. A WofE value of 13.95 was found in the elevation factor sub-class of >161m, which indicates a strong positive correlation between elevation and gully occurrence. The 0.96–1.83 slope subgroup has the maximum WofE value (6.101), which indicates a high positive correlation with the occurrence of gully erosion. In terms of the slope aspect, a high probability of gully occurrence is suggested by the north-east aspect with the WofE value of 4.8. The rainfall class of 781–797 mm with a value of 8.016 shows a strong relationship with gully erosion. The distance from river and lineament classes of 0–0.18 km and 0.19–0.43 km, with the WofE values of 4.054 and 3.703, demonstrate a strong inverse relationship with the occurrence of gullies. In the case of the geology and soil type, most of the gullies were found in the granite-gneiss geological formation and the fine loamy, mixed hyperthermic haplustepts soil type class. The WofE values of these classes are 3.408 and 11.625, indicating a high probability of gully occurrence. Generally, the LULC types strongly determine the development of gullies in a region [14]. For the LULC, the fallow land with the WofE value of 18.167 shows a strong correlation with the gully occurrence. The NDVI class of 0.16 to 0.20 with WofE value of 4.809, the TWI class of 9.73–11.85 with a WofE value of 3.55, the SPI class of 0.39–0.10 with a WofE value of 6.807, the STI class of 2.04–8.48 with a WofE value of 2.165 and the length of overland flow (lof) class of 1.92–2.27 with a WofE value of 7.861 represent strong and positive correlation with the occurrence of gullies.

### 3.3. Spatial Gully Erosion Susceptibility Analysis

**Figure 7:**
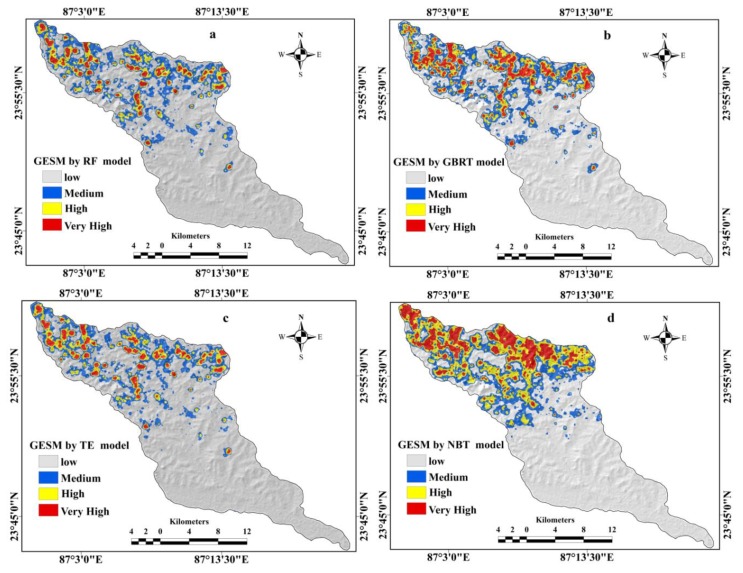
Gully erosion susceptibility maps (GESMs) showing (**a**) RF model, (**b**) GBRT model, (**c**) TE model, (**d**) NBT model.

The gully erosion models were built using ensemble machine learning algorithm-based training datasets to predict the spatial susceptibility to gully erosion. The gully erosion susceptibility (GES) indices produced by the machine learning techniques RF, GBRT, NBTree, and TE have been classified into four classes with respect to gully erosion susceptibility, namely low, medium, high, and very high, based on the natural break classification method. The Figure 7a–d show the GESMs produced by the four ensemble machine learning frameworks. 

The GESM produced by the RF model (Figure 7a) found that 2.29% (10.15 km^2^) of the basin area has a very high GES. The high and moderate GES zones cover 4.59% (20.34 km^2^) and 15.44% (68.38 km^2^) of the watershed, respectively (Table 4), while the remaining 344.07 km^2^ (77.68%) falls into the low gully erosion susceptibility class. The relative importance of the gully conditioning factors has also been assessed using the random forest method. According to this model, the elevation and rainfall are the most important contributing factors for gully erosion (Table 5), while the geology plays less of a role for gully erosion.

**Figure 8:**
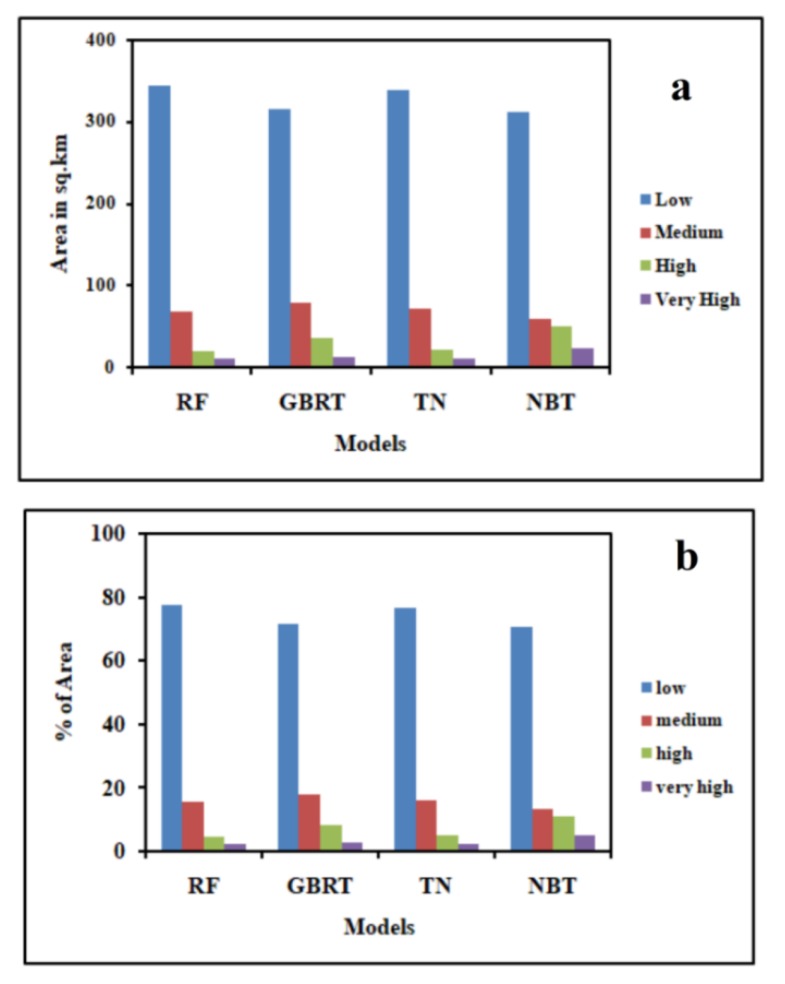
Graphs showing (**a**) areal distribution of RF, NBT, GBRT, and TE models, (**b**) distribution of the percentage of area of RF, NBT, GBRT, and TE models.

The GESM generated by the NBT (Figure 7d) shows that 70.45% (312.05 km^2^) of the study area has a low GES. The high and very high GES classes make up 11.13% and 5.21% of the watershed, respectively (Table 4 and Figure 8). The medium susceptibility class covers 58.49 km^2^ (13.20%) of the basin.

Based on the results of GBRT (Figure 7b), the research area has 315.85 km^2^ (71.31%) that falls into the low susceptibility class, followed by 78.47 km^2^ (17.71%) in the medium susceptibility class, 35.60 km^2^ (8.04%) in the high susceptibility class and 13.03 km^2^ (2.94%) in the very high GES classes (Table 4) In case of the TN model (Figure 7c), the results show that the study area has 338.66 km^2^ (76.46%) area in the low susceptibility class, followed by 71.76 km^2^ (16.20%) in the medium susceptibility class, 21.67 km^2^ (4.89%) in the high susceptibility class, and 10.86 km^2^ (2.45%) in the very high GES class out of the total area of 442.95 km^2^ (Table 4, Figure 8a,b). The relative importance of the GCFs was also assessed by the GBRT as like RF model. Similarly, elevation and rainfall are the most important factors while the geology is the least important factor contributing to gully occurrence (Table 5).

Fallow and barren land are extensively open to soil depletion by flowing water because of the absence of vegetation cover. Despite the complexities of gully formation, the main reason for it in this region is the intense monsoonal rainwater runoff. The region experiences a short rainy season with high-intensity precipitation events after hot and dry summers, which are ideal gully forming conditions. 

### 3.4. Validation of Models

**Figure 9:**
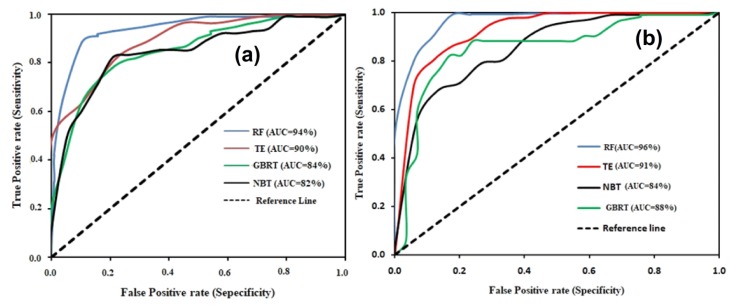
ROC curves showing AUC of RF, GBRT, TE, and NBT models (**a**) training dataset and (**b**) validation dataset.

**Figure 10:**
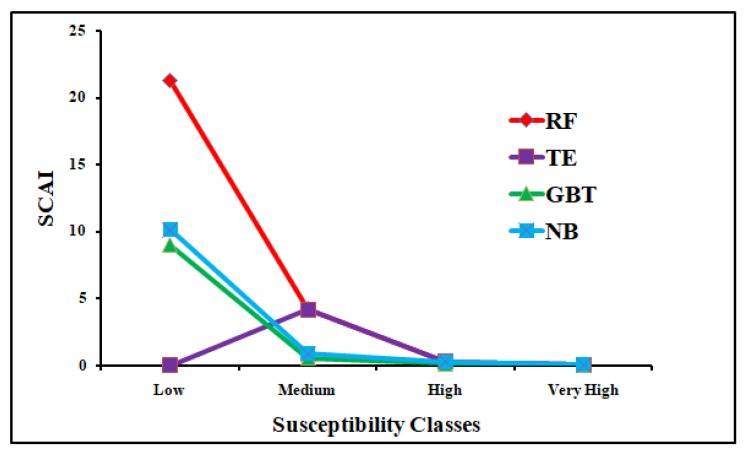
Seed cell area index (SCAI) values for different susceptibility classes in RF, NBT, GBRT, and TE models.

The validation of the GESMs using AUROC, SCAI, and eleven statistical measures are shown in Figure 9, Figure 10 and Table 6. In this research, we used some validation techniques that are rarely used in hazard modeling. To judge the capabilities of the models, we considered both the training and testing datasets. We found a good similarity between the data collected during fieldwork and the predicted results. Some field photos of gullies are presented in the methodology section. The success rates and predictive rates of the RF, TE, GBRT, and NBT models are 0.94, 0.90, 0.84, and 0.82 and 0.96, 0.91, 0.88, and 0.84, respectively. The AUCs of the AUROC indicate a very good to excellent prediction accuracy of the models for the GESMs (Table 6). 

The precision values for the training and validation datasets of the RF, TE, GBRT, and NBTree models are 0.98, 0.98, 0.80, and 0.93 and 1.00, 0.96, 0.43 and 0.85, respectively. The accuracy values for the training and validation datasets of the RF, TE, GBRT, and NBTree models are 0.87, 0.82, 0.80, 0.81 and 0.87, 0.91, 0.37, and 0.83, respectively. The MAE and RMSE values of the RF, TE, GBRT and NBTree models are 0.07, 0.23, 0.16, 0.18 and 0.15, 0.28, 0.29, and 0.33, respectively, for the training datasets. The MAE and MRSE values for the validation datasets for the RF, TE, GBRT, and NBTree models are 0.11, 0.25, 0.19, 0.23 and 0.19, 0.33, 0.31, and 0.35, respectively. The SCAI values decrease from low susceptibility classes to very high susceptibility classes, which indicates the more accurate and significant results (Table 6). The results of the seed cell area index (SCAI) for the very high susceptibility classes are 0.01 (RF), 0.03 (GBRT), 0.04 (NBT), and 0.01 (TE), which indicates that these are very good models (Table 6). All the machine learning methods used in this study are well suited for modeling the gully erosion susceptibility. According to the AUROC curve, the SCAI and all the statistical measures, the RF model is the most accurate and robust model for gully erosion prediction.

## 4. Discussion

Gully erosion risk assessment based on GESMs and effective geo-environmental factors is the first step for managing gully erosion. Although different approaches and procedures for the spatial prediction of environmental hazards have been developed and implemented around the world, the aims of all these methods are the same. A controversial issue among environmental researchers is the preparation of a logical and reliable susceptibility map of natural hazards. In the past decade, machine learning techniques are being developed. The important applications of the machine learning techniques are prediction, categorization, clustering, and elaboration of data [83,84]. Different sources were used to prepare the input dataset. Because some of the factors considered in the GESM were derived from a digital elevation model (DEM), the resolution of the DEM greatly affects the precision of the results [85,86]. In this study, we used RF, GBRT, NBT, and TE tree-based machine learning algorithms for producing the gully erosion susceptibility maps based on training and validation datasets and 14 GCFs. These factors were tested for collinearity by TOL and VIF. The results indicate that no GCF has a multi-collinearity problem. The outcomes of the WofE showed that the effective parameters and gully erosion datasets have a strong positive correlation. The positive values of the effective parameters indicate a strong correlation with the probability of gully erosion. As per the RF and GBRT, the most effective factors are elevation, rainfall, NDVI, LULC, and slope, while the geology, soil type and distance from the river have little control over gully erosion. In this basin, the geology, and soil type are almost uniform, which may be the cause of them having less impact on gully erosion. The GESMs based on machine learning ensemble techniques, namely the RF, NBTree, GBRT, and TE models, were created using GIS and R programming language. In the upper parts of Hinglo River basin, we identified a very high susceptibility class of GES. Geologically, the upper catchment consists of the granite-gneiss geological formation. The soil type of the upper catchment is the fine loamy mixed type soil textural. Topographically, the upper catchment is rugged and badland topography. The study area covers the two topographical regions of the Chhoto nagpur plateau and the Rar lateritic region. 

The AUROC, SCAI, and five statistical measures were used for validating the GESMs produced by the selected machine learning techniques and showed excellent accuracy in the prediction of gully erosion. For a number of reasons, it is not possible to completely eradicate or avoid some causes of errors, such as that the gully samples were chosen in an area where the gully erosion area is small in comparison to the non-gully area. We divided the sample data in a 70:30 ratio based on the suggestions in previous literature without testing the sample accuracy. A different ratio may yield better results, and that should be the subject of our future research. Noise in the selected gully conditioning factor data exists after the collinearity test and need other methods need to be applied to eradicate this problem. But the main advantage of the tree-based machine learning algorithms is in the collection of important information because they automate the process of investigating multiple datasets. The effective analysis of the non-gully area ratio, evaluation of sample division, considering the method for selecting the features, and use of ensemble approaches is useful in enhancing the accuracy of the GES models.

### Models Comparisons

In this study the results of these four models i.e., RF, GBRT, NBT, and TE were categorized into low, medium, high, and very high GES zones (Figure 7). The division of the area into different GES classes is shown in Figure 8a,b. Among these models, the Naïve Bayes tree (NBT) model shows the largest area of the very high susceptibility zone. According to the validation results, these models have proved the excellent prediction accuracy. But the results of these models vary slightly in terms of the values of the AUROC curve, SCAI and the eleven statistical measures (Table 6). The AUC of the RF model is 96%, which indicates this to be the most accurate, followed by 91% of TE, 88% of GBRT, 84% of NBT model based on the validation dataset. Therefore, the RF model is a better model for the prediction of GES (Table 6) for this basin compared to the other models. The findings also showed that the result of the tree-based ensemble methods has a better accuracy than the statistical models used in this region [23,24,87]. Our results are rational as the tree-based machine learning algorithms minimized bias, variance, and overfitting issues in GES modeling. This is confirmed by Arabameri et al. [88], Pourghasemi et al. [89], Hembram et al. [87], and Gayen et al. [90].

## 5. Conclusions

The purpose of this study is not only to investigate the capability of a machine learning model to predict the susceptibility to gully erosion, but also to compare its capability and robustness among the implemented models, i.e. GBRT, RF, NBT, and TE. Therefore, 14 geo-environmental factors were used and the significance of all GCFs was explored using the WofE, RF, and GBRT models. The findings underlined that the understanding of the strengths and limitations remains somewhat challenging for model selection, even when performing model comparisons with some clear objectives, such as prediction accuracy and robustness. Based on six threshold-dependent and -independent assessment criteria, the RF obtained the most outstanding performance as per the achievements. The GBRT, NBT, and TE have a slightly lower precision when compared to the RF in terms of pure prediction performance. The results of all the models show that the upper portion of the basin has the highest susceptibility to gully erosion in the whole basin. Therefore, immediate suitable planning is needed to prevent further gully and soil erosion in the Hinglo River basin. The outcome of variable significance showed that the elevation is the most significant GCF followed by the influences of rainfall and the NDVI. On the other hand, the geology, soil type, and STI influences are the least important. The results of this research could be helpful for land resource management to cope with the current uncertain situation and more accurately understand the different factors that influence gully erosion. Additionally, this approach could be used as a guideline for future research to analyze the vulnerability of gully erosion to land use change i.e., as a tool for regional soil resource analysis. 

## Figures and Tables

**Table 1:** Data sources.

Data Types	Sources	Scale	Year
Open series topographical map (73 M/1, 73M/5, 73M/6, 73P/4, 73L/13 and 73L/16)	Survey of India (SOI)	1:50,000	2011
Geological map (73/m)	Geological Survey of India (GSI)	1:50,000	1985
Soil type map	National Bureau of Soil Survey and Land Use Planning, Kolkata, collected in	1:50,000	2018
ASTER DEM	Earthexplor.usgs.gov, path = 139, Row = 43	30 × 30 m	2016
Landsat 8OLI/TIRS	Earthexplor.usgs.gov, path = 139, Row = 43	30 × 30 m	2018
Google Earth image	Data SIO, NOAA, U.S Navy, NGA, GEBCO	30 × 30 m	2018
Rainfall	Indian Meteorological Department (IMD)	Weather station data	Last three year

**Table 2:** Multi-collinearity analysis of the gully conditioning factors.

Conditioning Factors	Collinearity Statistics	
	Tolerance	VIF
Elevation	0.220	4.544
Slope	0.835	1.197
Aspect	0.899	1.112
Monsoonal rainfall	0.226	4.430
Geology	0.585	1.710
Soil type	0.492	2.034
Distance from River	0.621	1.609
Distance from Lineament	0.952	1.050
LULC	0.842	1.187
NDVI	0.793	1.260
TWI	0.844	1.185
STI	0.770	1.299
SPI	0.778	1.286
Length of overland flow (Lof)	0.608	1.644

**Table 3:** Relationship between gully erosion and gully condition factors using the WofE model.

Factors	$X^{+}$	$Y^{-}$	*p*	$Q^{2}X^{+}$	$Q^{2}Y^{-}$	Q(P)	$\frac{P}{Q(P)}$
**Elevation(m)**							
64–95	0.00	0.513	0.0	0.000	0.006	0.000	0.000
95–118	−2.44	0.402	−2.84	0.200	0.006	0.454	−6.255
118–138	0.733	−0.24	0.973	0.018	0.009	0.166	5.868
138–161	1.997	−0.34	2.337	0.020	0.009	0.171	13.698
161–284	2.082	−0.32	2.409	0.021	0.009	0.173	13.955
**Aspect**							
F	0.020	0.000	0.021	0.493	0.006	0.707	0.029
N	−1.16	0.02	−1.19	0.492	0.006	0.706	−1.690
NW	0.961	−0.10	1.064	0.042	0.007	0.221	4.818
E	0.245	−0.03	0.283	0.041	0.007	0.220	1.285
SE	−0.12	0.019	−0.14	0.049	0.007	0.238	−0.591
S	−0.13	0.024	−0.16	0.045	0.007	0.228	−0.704
SW	0.567	−0.11	0.686	0.028	0.008	0.189	3.637
W	−0.01	0.002	−0.01	0.045	0.007	0.228	−0.066
NW	−1.17	0.091	−1.26	0.164	0.006	0.413	−3.067
Slope (Degree)							
0–0.96	−0.49	0.340	−0.83	0.020	0.009	0.169	−4.907
0.96–1.83	0.583	−0.37	0.960	0.012	0.013	0.157	6.101
1.83–5.70	0.317	−0.05	0.374	0.035	0.008	0.206	1.819
5.73–12.85	0.000	0.073	0.000	0.000	0.006	0.000	0.000
12.85–24.65	0.000	0.005	0.000	0.000	0.006	0.000	0.000
Monsoonal Rainfall (mm)							
781–797	0.833	−0.42	1.262	0.013	0.012	0.157	8.016
767–781	0.489	−0.16	0.653	0.020	0.009	0.169	3.857
757–767	−0.28	0.078	−0.36	0.033	0.008	0.203	−1.803
748–757	−2.66	0.195	−2.85	0.466	0.006	0.688	−4.153
738–748	0.000	0.176	0.000	0.000	0.006	0.000	0.000
Soil Texture							
Sandy	0.000	0.062	0.000	0.000	0.006	0.000	0.000
Sandy Loam	0.001	0.000	0.001	0.388	0.006	0.628	0.002
Clay loam	0.000	0.971	0.000	0.000	0.006	0.000	0.000
Clay	0.000	0.018	0.000	0.000	0.006	0.000	0.000
Loam	0.000	0.037	0.000	0.000	0.006	0.000	0.000
Hapalustepts	1.903	−3.29	5.197	0.007	0.193	0.447	11.625
Paleustepts	−1.88	0.095	−1.98	0.387	0.006	0.627	−3.160
Geology							
Barakar formation	−1.69	0.028	−1.72	1.001	0.006	1.004	−1.720
Ironstone Shale	0.000	0.006	0.000	0.000	0.006	0.000	0.000
Quartzite	0.000	0.002	0.000	0.000	0.006	0.000	0.000
Granite	0.167	−3.25	3.419	0.006	1.000	1.003	3.408
Newer Alluvium	0.000	0.124	0.000	0.000	0.006	0.000	0.000
Land Use/Land Cover							
Water bodies	0.000	0.020	0.000	0.000	0.006	0.000	0.000
Settlement	0.000	0.044	0.000	0.000	0.006	0.000	0.000
Natural Vegetation	0.000	0.107	0.000	0.000	0.006	0.000	0.000
Fallow land	2.375	−0.54	2.916	0.015	0.011	0.161	18.167
Agricultural land	−0.35	0.767	−1.12	0.011	0.014	0.158	−7.073
NDVI							
0.15–0.16	−0.88	0.169	−1.05	0.062	0.007	0.263	−4.002
0.16–0.20	0.524	−0.24	0.770	0.015	0.010	0.160	4.809
0.20–0.23	0.598	−0.19	0.794	0.019	0.009	0.168	4.727
0.23–0.28	0.039	−0.00	0.045	0.043	0.007	0.223	0.202
0.28–0.43	−2.08	0.201	−2.29	0.245	0.006	0.501	−4.568
Distance from River (km)							
0–0.18	0.312	−0.33	0.650	0.010	0.015	0.160	4.054
0.18–0.42	0.182	−0.08	0.269	0.017	0.010	0.164	1.638
0.42–0.72	−1.25	0.129	−1.38	0.133	0.006	0.374	−3.696
0.72–1.16	0.000	0.089	0.000	0.000	0.006	0.000	0.000
1.16–2.08	0.000	0.020	0.000	0.000	0.006	0.000	0.000
Distance from Lineament (km)							
0–0.19	−0.34	0.184	−0.52	0.021	0.009	0.173	−3.037
0.19–0.43	0.346	−0.23	0.583	0.013	0.012	0.157	3.703
0.43–0.69	0.278	−0.06	0.342	0.029	0.008	0.191	1.789
0.69–0.99	−1.48	0.055	−1.53	0.399	0.006	0.637	−2.415
0.99–1.65	0.000	0.021	0.000	0.000	0.006	0.000	0.000
Sediment Transport Index (STI)							
0–2.04	0.010	−0.09	0.100	0.007	0.068	0.273	0.365
2.04–8.48	0.551	−0.04	0.593	0.068	0.007	0.274	2.165
8.48–20.76	0.000	0.041	0.000	0.000	0.006	0.000	0.000
20.76–42.99	0.000	0.006	0.000	0.000	0.006	0.000	0.000
42.99–74.59	0.000	0.001	0.000	0.000	0.006	0.000	0.000
Topographic Wetness Index (TWI)							
2.92–7.35	−0.42	0.076	−0.49	0.051	0.007	0.240	−2.078
7.35–8.38	−0.23	0.120	−0.35	0.021	0.009	0.172	−2.071
8.38–9.73	0.112	−0.05	0.162	0.019	0.009	0.168	0.962
9.73–11.85	0.567	−0.11	0.680	0.029	0.008	0.191	3.555
11.85–19.30	0.478	−0.01	0.494	0.153	0.006	0.399	1.240
1.47–0.88	0.000	0.002	0.000	0.000	0.006	0.000	0.000
0.88–0.39	0.000	0.003	0.000	0.000	0.006	0.000	0.000
0.39–0.10	1.339	−0.13	1.470	0.039	0.007	0.216	6.807
0.10–0.03	−0.07	0.020	−0.09	0.029	0.008	0.191	−0.473
0.03–0.42	−0.14	0.299	−0.44	0.010	0.017	0.162	−2.729
Length of Overland Flow (sq. km)							
0–1.42	1.182	−0.08	1.269	0.054	0.007	0.247	5.146
1.42–1.92	0.838	−0.10	0.938	0.040	0.007	0.217	4.320
1.92–2.27	0.964	−0.31	1.280	0.017	0.010	0.163	7.861
2.27–2.58	−0.08	0.035	−0.11	0.022	0.009	0.175	−0.677
2.58–2.89	−1.83	0.520	−2.35	0.086	0.007	0.304	−7.741

**Table 4:** Seed cell area index (SCAI) values of RF, NBT, GBRT, and TE models.

Models	Classes	Total Area (km^2^)		Testing Gullies (km^2^)		Validation Gullies (km^2^)		SUM	SCAI
		Area (km^2^)	% of Area	Area (km^2^)	% of Area	Area (km^2^)	% of Area		
RF	Low	344.07	77.68	0.05	3.66	0.00	0.00	3.66	21.23
	Medium	68.38	15.44	0.05	3.66	0.00	0.00	3.66	4.22
	High	20.34	4.59	0.19	14.63	0.05	6.25	20.88	0.22
	Very High	10.15	2.29	0.99	78.05	0.73	93.75	171.80	0.01
TN	Low	338.66	76.46	0.00	0.00	0.00	0.00	0.00	0.00
	Medium	71.76	16.20	0.05	3.90	0.00	0.00	3.90	4.16
	High	21.67	4.89	0.13	10.39	0.07	8.93	19.32	0.25
	Very High	10.86	2.45	1.09	85.71	0.71	91.07	176.79	0.01
GBT	Low	315.85	71.31	0.03	2.60	0.04	5.36	7.95	8.96
	Medium	78.47	17.71	0.25	19.48	0.11	14.29	33.77	0.52
	High	35.60	8.04	0.36	28.57	0.30	39.29	67.86	0.12
	Very High	13.03	2.94	0.63	49.35	0.32	41.07	90.42	0.03
NB	Low	312.05	70.45	0.06	4.88	0.02	2.08	6.96	10.12
	Medium	58.49	13.20	0.09	7.32	0.06	8.33	15.65	0.84
	High	49.32	11.13	0.29	23.17	0.15	18.75	41.92	0.27
	Very High	23.10	5.21	0.82	64.63	0.55	70.83	135.47	0.04

**Table 5:** Relative influence of effective conditioning factors.

Effective Factors	Relative Influence by GBRT	Mean Decrease Accuracy by RF
Elevation	14.41	203.52
Monsoonal Rainfall	11.87	137.67
NDVI	7.13	136.52
LULC	6.87	123.95
Slope	5.07	100.28
SPI	4.01	99.39
Aspect	3.39	97.96
Length of overland flow (Lof)	2.14	82.60
Distance from lineament	1.96	75.24
TWI	1.96	65.49
Distance from River	1.59	57.54
STI	0.92	55.71
Soil type	0.68	42.93
Geology	0.23	4.61

**Table 6:** Performances of RF, GBRT, NBT, and TE models.

MODEL	Training Datasets				Validation Data Sets			
	RF	TE	GBRT	NBTree	RF	TE	GBRT	NBTree
Precision (PPV)	0.98	0.98	0.80	0.93	1.00	0.96	0.43	0.85
False discovery rate (FDR)	0.02	0.02	0.20	0.07	0.00	0.204	0.63	0.15
Accuracy	0.87	0.82	0.80	0.81	0.87	0.91	0.37	0.83
AUROC	0.94	0.90	0.84	0.82	0.96	0.91	0.88	0.84
MAE	0.07	0.23	0.16	0.18	0.11	0.25	0.19	0.23
RMSE	0.15	0.28	0.29	0.33	0.19	0.33	0.31	0.35
